# Effects of 5 Nanosilica Concentrations and Humid Environments at 6 Different pH Levels on Fracture Toughness and Moisture Absorption of Dental Polymethyl Methacrylate Resin‎ Reinforced With Silica Nanoparticles: An Explorative Experimental Scanning Electron Microscopy Study

**DOI:** 10.1002/cre2.70006

**Published:** 2024-12-03

**Authors:** Mohammad Ali Golshokouh, Nima Refahati, Pouyan Roodgar Saffari

**Affiliations:** ^1^ Department of Mechanical Engineering Damavand Branch Islamic Azad University Damavand Iran; ^2^ Department of Civil Engineering Thammasat School of Engineering, Faculty of Engineering ‎Thammasat University Pathumthani Thailand

**Keywords:** dental materials, fracture resistance, moisture absorption, nanomaterials, nanosilica, polymethyl methacrylate (PMMA), scanning electron microscopy, silicon nanoparticles

## Abstract

**Introduction:**

No study has assessed the effects of nanosilica within polymethyl methacrylate (PMMA) resin and environmental pH on resin's fracture resistance and moisture absorption.

**Methods:**

A total of 90 specimens were divided into 30 subgroups of three, as per the ASTM D5045 standard: five groups of nanosilica percentages (0%/2%/5%/7%/10%), each ‎divided into six subgroups of pH levels (pH = 5/6/7/8/9, + “dry” control). The specimens were prepared by mixing silica nanoparticles with PMMA powder in a vacuum mixer. Then, the specimens were mixed with a diluent liquid (TEGDMA) according to the manufacturer's instructions. For each of the five weight percentages, 36 samples were produced. The 18 specimens in each group were randomly divided into six subgroups of pH levels. The specimens were kept in containers of liquid at different pH levels at room temperature for 1 week. Their before‐ and after‐storage weights were recorded to calculate moisture absorption. The fracture resistance test was performed (ASTM D5045 standard) using the three‐point bending method. Scanning electron microscopy was performed. Data were analyzed.

**Results:**

Both nanosilica extents and pH levels significantly affected the fracture toughness with a significant interaction (*p* < ‎‎0.00001). All post hoc comparisons of different pH levels (except pH= 5 vs. 6) were significant (*p* < ‎‎0.0001). All post hoc comparisons of different nanosilica concentrations were significant (*p* < ‎‎0.0001). Both nanosilica extents and pH levels significantly influenced the fracture toughness with a significant interaction (*p* < ‎‎0.00001). All post hoc comparisons of different pH levels and also between different nanosilica concentrations were significant (*p* < ‎‎0.0001). The correlation between moisture absorption and fracture toughness was significant (*R* = −0.382, *p* = 0.0009).

**Conclusions:**

Fracture toughness decreases when placed in humid and acidic environments. Also, the samples that were placed in a humid environment suffered a brittle fracture. Increasing silica nanoparticles improved fracture toughness (becoming optimal at 5 wt% nanosilica).

**Objective and Materials:**

The objective of this study was to investigate the fracture toughness of dental samples made of PMMA reinforced with various percentages of nanosilica at various pH levels. For this purpose, dental resins with various amounts of nanosilica were placed in moist media at different pH values ranging from 5 to 9 (mimicking the normal pH range of the human mouth).

1

The most common material used in removable dental prostheses is polymethyl methacrylate (PMMA) resin. It is a glassy and brittle material that has low fracture resistance. Any dental restorative or prosthetic material, as well as natural teeth, must have sufficient mechanical integrity to function in the oral cavity for an extended period of time (Ilie et al. 2017). As removable dental prostheses are exposed to long‐term pressures in the oral cavity, it is important to improve their mechanical properties. The life of dental prostheses is short due to the damage caused by acid‐producing bacteria and breakage, as 63%−68% of dental prostheses break within a few years after their first use due to high chewing forces or accidents (Alhotan et al. 2021a). Therefore, resins used in dental prostheses should have considerable impact resistance and be able to withstand high‐impact forces without cracking (Alhotan et al. 2021a). To solve this problem, researchers have found various methods to improve the mechanical properties of this type of resin, including the use of fillers in the polymer phase.

Nanoparticles (NPs) have a very high surface‐to‐volume ratio, which increases molecular interactions between polymers and NPs in dental resins (Jiangkongkho, Arksornnukit, and Takahashi 2018). The properties of dental resins reinforced with nanofillers depend on the size, shape, type, and concentration of added particles. Various NPs such as ZrO_2_, SiO_2_, TiO_2_, and GO and also diamond NPs have been used to increase the physical and mechanical properties of PMMA (Cahyanto et al. 2023; Alhotan et al. 2021b). A review has summarized the incorporation of numerous nano‐filled materials into PMMA (An et al. 2023); the incorporated materials were shown to have positive antimicrobial effects against *Candida albicans*, *Staphylococcus aureus*, and *Streptococcus mutans* (An et al. 2023).

In recent years, silica NPs have attracted a lot of attention due to their regular structure, ease of surface modification, and affordable costs (Lee and Yoo 2016). NPs are often used as fillers to improve the properties of dental resin, including their wear resistance, mechanical properties, and refractive index. The strength of the chemical bond between PMMA and NPs is very important because it enables the dental resin material to transfer external forces from the weak polymer base to the much stronger NP fillers. Also, the homogeneous and uniform distribution of NPs in the polymer matrix reduces the formation of stress concentration areas and, as a result, improves the mechanical properties and light transparency of the dental resin (Fatalla, Tukmachi, and Jani 2020).

A coupling agent is used to improve the intermediate chemical bond between mineral materials (the base polymer) and organic materials (fillers) and forms a bridge at the interface of the two components (Elshereksi, Muchtar, and Azhari 2021). The use of silane coupling agents is a common method in the manufacture of dental resin, as they are more efficient, spread more easily throughout the dental resin, and have excellent chemical resistance (Aldabib 2021). In this regard, the use of gamma‐methacryloxypropyltrimethoxysilane (γ‐MPS) as a coupling agent significantly increases the tensile strength, bending strength, and hardness of dental resins.

It is diluted and dissolved in ethanol and water and the pH is adjusted to ca.2–6 for hydrolysis (activation) (Matinlinna, Lung, and Tsoi 2018).

The fracture toughness parameter of a material shows the resistance of that material against crack propagation. Materials with a low fracture toughness easily crack and fail. Hence, investigating the fracture toughness of dental resins is very important. The mechanical behavior of PMMA and SiO_2_ multilayer resins has been assessed by a few controversial studies, each assessing a different aspect of the matter (Fatalla, Tukmachi, and Jani 2020; Zhen et al. 2020; Balos et al. 2020; Siot et al. 2019; Mussatto et al. 2020; Salman, Jani, and Fatalla 2017; Topouzi et al. 2017; Kundie, Azhari, Muchtar, et al. 2018).

When it comes to physical and mechanical properties of dental resins, some important areas have not yet been studied; these include the percentage of filler particles and the environment to which the material is exposed. Especially in the case of dental resins, there is no information about the relationship between properties and structure, and between the acidic or alkaline environments to which the resin is exposed. Although studies have been conducted in the field of NPs‐enhanced resins and their physical ‎and mechanical properties, in these studies, some important factors affecting the mechanical properties ‎of these materials have been ignored, such as the environment to which the material is exposed. ‎Besides, in the case of NPs‐enhanced resins used in dentistry (dental resins), there is not much ‎information about the relationship between mechanical properties and microscopic structure. ‎Moreover, the acidic or alkaline environments that the resin is exposed to have ‎not been studied ‎ (Ilie et al. 2017; Elshereksi, Muchtar, and Azhari 2021; Siot et al. 2019; Mussatto et al. 2020; Salman, Jani, and Fatalla 2017; Topouzi et al. 2017; Kundie, Azhari, Muchtar, et al. 2018; Karabela and Sideridou 2011).

Another critical determinant of the physicomechanical aspect of dental resins is water sorption. Absorbed water reduces mechanical properties such as hardness, transverse strength, and fatigue limit due to its lubricating effect (Zirak et al. 2019). It also causes volume expansion and changes the occlusal surface of the denture (Ristic and Carr 1987). Changing the dimensions exposes the dental resin to internal stresses and then causes cracks and failures in the denture (Rajaee, Vojdani, and Adibi 2014). Therefore, it is important to study the effect of the environment to which these samples are exposed. Nonetheless, few studies are available concerning water sorption of nanosilica particles. Karabela and Sideridou (2011) observed that the amount of absorbed water increases by decreasing the size of filler particles.

Due to the above‐mentioned numerous gaps in the literature, and because of the clinical importance of this subject, this is the first explorative study to be conducted. The goal was to investigate the fracture toughness of dental samples made of PMMA reinforced with various percentages of nanosilica under various pH levels. For this purpose, dental resins with various amounts of nanosilica were placed in moist media at different pH values ranging from 5 to 9 (mimicking the normal pH range of the human mouth). As detailed above, the novelty of this research is that by simulating the environment in which the dental resin is placed, the effects of ‎humidity and acidity on the microscopic structure and the fracture toughness ‎‎(as major factors in the failure of dentures) (Alhotan et al. 2021a) were quantitatively and microscopically examined‎. The fracture toughness of PMMA was examined using the three‐point bending method, which simulates more accurately the stress applied to the denture resin during mastication (Elshereksi, Muchtar, and Azhari 2021). The combination of the effects of nanosilica percentage and pH on reinforced PMMA was assessed statistically. The null hypotheses were (1) lack of any difference among fracture toughness values of different nanosilica percentages, (2) lack of such differences among different pH values, and (3) lack of any interactions between pH values with nanosilica percentages. Besides quantitative analyses, scanning electron microscopy (SEM) was performed as well in order to examine the fractures and surfaces accurately.

## 2 Materials and Methods

This was an explorative in vitro experimental study. As it did not involve any humans, animals, or life forms, the need for any consent was waived by the Research Committee of the University. The study protocol and its ethics were approved by the Research Committee of the Islamic Azad University (Thesis Code: 352648000617703105008162424173).

### 2.1 Materials and Equipment in Use

In this experimental laboratory study, PMMA was used as a denture base material (Taiwan Chemical Company, Taiwan) with a specific gravity of 1.19 g/cm^3^. The process of polymerization and production of PMMA were carried out at the Razi Metallurgy Research Institute in Iran and the production process was carried out under the petrochemical license of Taiwan. Silica NPs with a size of 20 nm and a purity of 99.5% (Acrosun, Spain) were used as base polymer reinforcements. To reduce the viscosity, TEGDMA thinning resin (Evonic, Germany) was applied. Molding of the samples was performed using a two‐piece aluminum mold with dimensions of 6 × 4 × 50 mm. To prevent the samples from sticking inside the mold, an acrylic separating fluid (Major, Italy) was used. Also, to modify the surface of silica NPs, the acrylate silane modifying agent, γ‐MPS, was used (Sigma Aldrich, Germany) as it is the most widely used silane coupling agent in dental resins (Karkanis et al. 2021; Kundie, Azhari, and Ahmad 2018; Rossi Canuto de Menezes et al. 2021).

To measure the weight of the samples, a laboratory scale at 0.001 g accuracy (model M1003i, Bel, Italy) was used. An STM250 tensile device (Centam, England) was used to measure the fracture toughness of the samples. A mechanical stirrer (blender type) with a maximum power of 2000 revolutions per minute was applied to combine silica NPs with the base polymer. To measure the pH of the environment, the pH meter model PH‐80, manufactured by HM Digital Company, was used.

### 2.2 Sample Production

#### 2.2.1 Nanosilica

In order to achieve a uniform composition of silica NPs and PMMA, a vacuum mixer was used.

In order to reduce the formation of agglomerates and achieve a better distribution of the filler particles inside the matrix, the surface of silica NPs was modified using an acrylate silane agent, ϒ‐MPS, and the hydrolysis–condensation method. For this purpose, a solution of water and alcohol with a weight ratio of 70 to 30 was used for the hydrolysis of ϒ‐MPS for 30 min. The pH was adjusted to around 3−4 by acetic acid. Then, NPs were added to this solution and kept at room temperature for 1 week. The NPs were washed with ethanol and then in room air, the remaining alcohol was evaporated, and the NPs were dried.

#### 2.2.2 Incorporation of Nanosilica Into PMMA

To produce each desired combination of nanosilica‐incorporated PMMA resin specimens, first, modified silica NPs with specific weight percentages (0% [control], 2%, 5%, 7%, and 10%) were mixed with polymer methyl methacrylate (PMMA) powder in a mixer. Grinding was performed for 12 min at a frequency of 20 Hz and a speed of 100 rpm.

To produce nanomaterial‐enhanced resin specimens, the mixture of methyl methacrylate powder and milled NPs was mixed with a diluent liquid (TEGDMA) according to the manufacturer's instructions. This process was carried out mechanically at room temperature using a small stirrer.

#### 2.2.3 Preparation of Each Specimen

To prevent the produced paste from sticking to the mold, the mold surface was covered with a layer of acrylic separating liquid. Once the surface of the mold was dried (after about 3 h), the paste‐like liquid was poured into each part of the mold; then, the two parts of the mold were secured together. Next, the mold was immersed in hot water at a constant temperature of 80°C for 50 min. After that, the mold was removed and cooled down to room temperature, and then the polymerized sample was removed from the mold. Afterward, the samples were polished first with a sandpaper with 600 grit and then with a sandpaper with 1000 grit.

### 2.3 Grouping

#### 2.3.1 Sample Size

There were a total of 30 subgroups in this study: five groups of nanosilica percentages, each divided into six subgroups of pH levels. The sample size was determined as three specimens per subgroup (as per the ASTM D5045 standard) totaling 90 specimens in general.

#### 2.3.2 Grouping Based on Nanosilica Percentage

Initially, for each of the five weight percentages of nanosilica (i.e., 0%, 2%, 5%, 7%, 10%), 50 samples were produced. They were inspected carefully, and for each of the five groups, the best 15 samples with the least number of defects, the smoothest surfaces, and the fewest bubbles were selected. The group with zero percent of nanosilica was the control. The groups were named A (0% nanosilica, control), B (2%), C (5%), D (7%), and E (10%).

#### 2.3.3 Grouping Based on pH

There were six groups of pH. Considering that the pH range of a healthy adult human mouth is 5−9, there were five groups of pH, with pH = 5, 6, 7, 8, and 9. A “dry” control group was included as well, in which there was no moisture in the environment.

The 18 specimens in each of the above five nanosilica groups were randomly divided into six subgroups of pH (*n* = 5 nanosilica groups × 6 pH groups). One of these subgroups (in each of the five groups of nanosilica, *n* = 5 × 3) was the control, in which the samples were not placed in a humid environment. The rest of the samples were divided randomly into the remaining five pH groups as mentioned above (*n* of each = 3 samples in each subgroup, 25 subgroups, overall *n* = 75). The samples were named as shown in Table 1.

**Table 1:** Naming of the 30 subgroups (*n* = 30 subgroups × 3 specimens per subgroup).

Nanosilica	pH					
	Dry control	5	6	7	8	9
A (0% sio_2_)	A0	A5	A6	A7	A8	A9
B (2% sio_2_)	B0	B5	B6	B7	B8	B9
C (5% sio_2_)	C0	C5	C6	C7	C8	C9
D (7% sio_2_)	D0	D5	D6	D7	D8	D9
E (10% sio_2_)	E0	E5	E6	E7	E8	E9

### 2.4 Moisture Absorption Test

All the specimens, except the ones within the dry environment (subgroups A0 to E0 in Table 1), were placed in closed containers with a pH of 5−9 for 1 week.

The moisture absorption test was performed under the ASTM D570 standard. As the pH range of a healthy and adult human is between 5 and 9, for this purpose, we performed moisture absorption tests in this range (at pH values of 5, 6, 7, 8, and 9). Sealed containers were used to increase the contact surface of the samples with the liquid and also to exclude any potential effects of the environment humidity or temperature on the pH of the tested liquid. Thus, each sample was placed in a coded container. The duration of the test for the moisture absorption test was 7 days. The samples were immersed in the solution for a week.

For pH = 7, we used the same distilled water, the pH of which was measured with a device. ‎The liquid with different pH was also purchased from Pishgaman Shimi Company. These were distilled water that had been acidified using phosphoric acid or alkalinized using sodium bicarbonate. The pH of the solution was measured and calibrated in the laboratory of the Dezful Standard Department using a P811 pH meter with an accuracy of 0.1 (Yoko Company).

During the tests, the liquid bath was in temperature equilibrium with the environment at a temperature of 20 ± 2°C. A digital scale with an accuracy of 0.001 g (M1003i, Bel, Italy‎) was used to weigh the samples. The amount of moisture absorption was determined by measuring the weight of the samples before and after placing them in the liquid bath according to the following ASTM D570 standard‎ formula (Equation 1):

(1) $$\frac{\Delta w}{W}=\frac{M2-M1}{M1}$$

where *M1* is the primary mass of the sample and *M2* is the secondary mass of the sample.

### 2.5 Fracture Resistance Test

**Figure 1:**
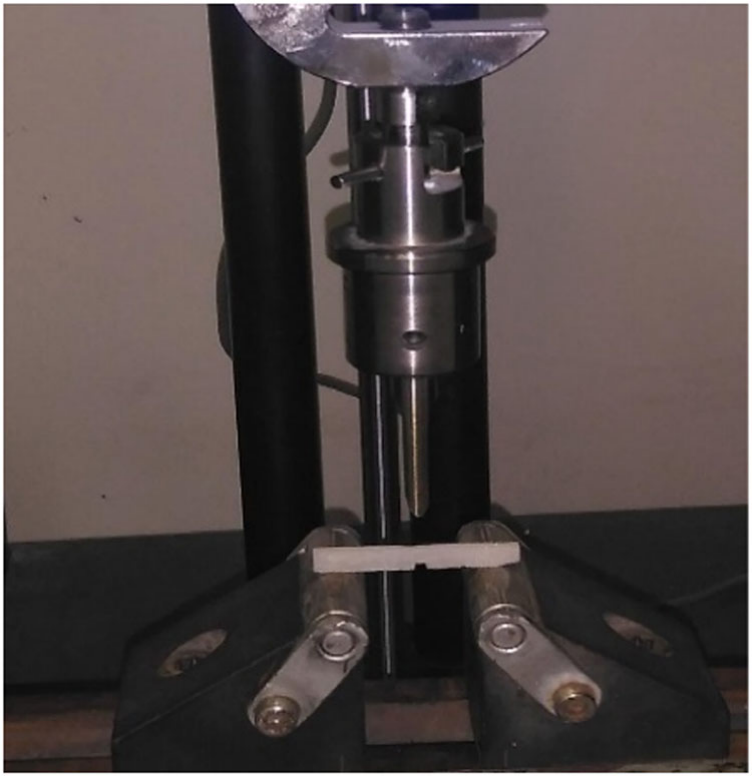
A sample tested in the bending test machine.

The fracture resistance test of nanosilica‐enhanced resin was performed based on the ASTM D5045 standard and using the three‐point bending method. In this test, a universal testing machine (AL‐7000‐M, Gotech, Taiwan) with a speed of 1.0 mm/min and a load cell of 20 KN was used. The method of loading the resin is shown in Figure 1, in which a rectangular piece with an edge crack of length is subjected to a three‐point bending load.

Before conducting the fracture toughness test, according to the standard, prefabricated rectangular pieces were cut using a thin saw blade with a thickness of 250 mm to create an initial crack in the samples. The notches created by the thin blade were sharpened by pressing a razor. In this way, the grooves created in the samples can be assumed to have acceptable accuracy as a sharp crack to perform the fracture toughness test. Finally, the length of the edge crack created in the middle of each sample is equal to 3 mm. As a result, the crack length ratio to width of the sample in all experiments of this research is a constant value of 0.5.

The fracture toughness test was performed on all of the 90 specimens described in Table 1. Of these, 75 specimens were placed in closed environments with different pH values for 1 week. In both groups of samples, before performing the bending test, the samples were removed from the containers and kept for 24 h in the standard conditions of the laboratory environment (temperature: 23 ± 2°C, humidity: 50 ± 5%).

Then, the cracked samples were placed inside the supports of the three‐point bending fixture (with a support distance of 40 mm) in the universal testing machine and were monotonically loaded at a constant rate of 1 mm/min (Figure 4). Loading continued until the moment of crack growth from the initial crack tip and complete failure of the parts, and finally, the load–displacement curve of each sample was obtained. Using the critical load corresponding to the fracture moment of each sample (*F*_S_), the value of fracture toughness (*K*_IC_) of dental resin specimens was calculated using Equation 2.

(2) $$\;K_{\mathrm{IC}}=f\left(\frac{a}{w}\right)\frac{F_{S}}{t\sqrt{w}}\times \mathrm{MPa}m^{\frac{1}{2}}$$

where *f*(*a*/*w*) is the geometric coefficient, and it depends on the type of part and applied loading. This coefficient was calculated using finite element modeling of the tested part for the conditions of *s*/*w* = 3.3 and *a*/*w* = 0.5. Also, *t* is the thickness of the sample (in millimeters) and *w* is the width of the sample (in millimeters) (Equation 3).

(3) $$f\left(\frac{a}{w}\right)=\frac{\left(2+\frac{a}{w}\right)(0.886+4.64\frac{a}{w}-13.32\frac{a^{2}}{w^{2}}+14.72\frac{a^{3}}{w^{3}}-5.6\frac{a^{4}}{w^{4}})}{{\left(1-\frac{a}{w}\right)}^{\frac{1}{2}}}$$

### 2.6 SEM

After performing the fracture toughness test, we evaluated the fracture section of the samples ‎using an electron microscope (VEGA/TESCAN‐XMU model, the Czech Republic) at different magnifications. The ‎samples were selected based on two factors: the highest amount of moisture absorption and ‎the highest amount of fracture toughness. All samples were placed at pH = 7 and all samples had 5% ‎filler.‎ To perform the SEM test, the samples were first placed in a dehumidifier and completely dried. The samples were coated with gold according to the laboratory standard RMRC‐WI‐550‐103‐01. Afterward, electron micrographs were taken from the samples at magnifications of ×200, ×1000, and ×3000. The details of SEM images, including the voltage, field of view, depth, and magnification, are specified below each image.

### 2.7 Statistical Analysis

Descriptive statistics and 95% confidence intervals (CIs) ‎were calculated. Two‐way analysis of variance (ANOVA) and the Bonferroni post hoc test of SPSS 25 (IBM, Armonk, NY, USA) were used to evaluate the effects of nanosilica concentrations and environment pH on moisture absorption and fracture toughness. The Pearson's correlation coefficient was used to evaluate the correlation between moisture absorption and fracture toughness. The level of significance was predetermined as 0.05.

## 3 Results

The raw results pertaining to moisture absorption of all 75 specimens are presented in Table 2. Overall, the mean and standard deviation (SD) for moisture absorption in all 75 samples were ‎0.0149880 ± ‎0.01249969‎ (standard error: ‎0.00144334, 95% CI: ‎0.0121121 to ‎0.0178639, minimum: ‎0.00140‎, maximum: ‎0.06460), respectively. ‎Descriptive statistics and 95% CIs for moisture absorption in different groups and subgroups are presented in Tables 3 and 4. Raw results of fracture resistance are presented in Table 5. Overall, the mean and SD for moisture absorption in all 90 samples were ‎25.3506 ± 22.47070‎ (standard error: 2.40911, 95% CI: ‎20.5614 to ‎30.1397, minimum: ‎9.54‎, maximum: ‎107.40), respectively. ‎Descriptive statistics and 95% CIs for fracture resistance in different groups and subgroups are presented in Tables 6 and 7.

**Table 2:** Raw data for moisture absorption.

Nanosilica	pH				
	5	6	7	8	9
A (0%)	0.0176	0.0237	0.0641	0.0198	0.0177
	0.0174	0.0236	0.0646	0.0190	0.0175
	0.0173	0.0244	0.0644	0.0199	0.0178
B (2%)	0.0150	0.0192	0.0306	0.0094	0.0075
	0.0151	0.0196	0.0316	0.0095	0.0075
	0.0155	0.0197	0.0309	0.0095	0.0075
C (5%)	0.0149	0.0191	0.0183	0.0094	0.0071
	0.0148	0.0189	0.0192	0.0092	0.0070
	0.0141	0.0186	0.0192	0.0091	0.0070
D (7%)	0.0118	0.0192	0.0169	0.0087	0.0065
	0.0124	0.0185	0.0164	0.0087	0.0067
	0.0126	0.0186	0.0168	0.0087	0.0066
E (10%)	0.0023	0.0014	0.0058	0.0046	0.0032
	0.0022	0.0014	0.0058	0.0044	0.0034
	0.0023	0.0014	0.0061	0.0047	0.0032

**Table 3:** Descriptive statistics and 95% confidence intervals for moisture absorption in different nanosilica and pH groups.

Variable	Groups	*N*	Mean	SD	SE	95% CI		Minimum	Maximum
Nanosilica	A (0%)	15	0.0285867	0.01867507	0.00482188	0.0182448	0.0389286	0.01730	0.06460
	B (2%)	15	0.0165400	0.00869284	0.00224448	0.0117261	0.0213539	0.00750	0.03160
	C (5%)	15	0.0137267	0.00505618	0.00130550	0.0109266	0.0165267	0.00700	0.01920
	D (7%)	15	0.0126067	0.00477545	0.00123302	0.0099621	0.0152512	0.00650	0.01920
	E (10%)	15	0.0034800	0.00166442	0.00042975	0.0025583	0.0044017	0.00140	0.00610
pH	5	15	0.0123533	0.00549699	0.00141932	0.0093092	0.0153975	0.00220	0.01760
	6	15	0.0164867	0.00805542	0.00207990	0.0120257	0.0209476	0.00140	0.02440
	7	15	0.0273800	0.02085248	0.00538409	0.0158323	0.0389277	0.00580	0.06460
	8	15	0.0103067	0.00514080	0.00132735	0.0074598	0.0131535	0.00440	0.01990
	9	15	0.0084133	0.00503216	0.00129930	0.0056266	0.0112001	0.00320	0.01780

Abbreviations: CI, confidence interval; SD, standard deviation; SE, standard error.

**Table 4:** Descriptive statistics and 95% CIs for moisture absorption in different subgroups.

Nanosilica	pH	*N*	Mean	SD	SE	95% CI		Minimum	Maximum
A (0%)	5	3	0.0174333	0.00015275	0.00008819	0.0170539	0.0178128	0.01730	0.01760
	6	3	0.0239000	0.00043589	0.00025166	0.0228172	0.0249828	0.02360	0.02440
	7	3	0.0643667	0.00025166	0.00014530	0.0637415	0.0649918	0.06410	0.06460
	8	3	0.0195667	0.00049329	0.00028480	0.0183413	0.0207921	0.01900	0.01990
	9	3	0.0176667	0.00015275	0.00008819	0.0172872	0.0180461	0.01750	0.01780
B (2%)	5	3	0.0152000	0.00026458	0.00015275	0.0145428	0.0158572	0.01500	0.01550
	6	3	0.0195000	0.00026458	0.00015275	0.0188428	0.0201572	0.01920	0.01970
	7	3	0.0310333	0.00051316	0.00029627	0.0297586	0.0323081	0.03060	0.03160
	8	3	0.0094667	0.00005774	0.00003333	0.0093232	0.0096101	0.00940	0.00950
	9	3	0.0075000	0.00000000	0.00000000	0.0075000	0.0075000	0.00750	0.00750
C (5%)	5	3	0.0146000	0.00043589	0.00025166	0.0135172	0.0156828	0.01410	0.01490
	6	3	0.0188667	0.00025166	0.00014530	0.0182415	0.0194918	0.01860	0.01910
	7	3	0.0189000	0.00051962	0.00030000	0.0176092	0.0201908	0.01830	0.01920
	8	3	0.0092333	0.00015275	0.00008819	0.0088539	0.0096128	0.00910	0.00940
	9	3	0.0070333	0.00005774	0.00003333	0.0068899	0.0071768	0.00700	0.00710
D (7%)	5	3	0.0122667	0.00041633	0.00024037	0.0112324	0.0133009	0.01180	0.01260
	6	3	0.0187667	0.00037859	0.00021858	0.0178262	0.0197071	0.01850	0.01920
	7	3	0.0167000	0.00026458	0.00015275	0.0160428	0.0173572	0.01640	0.01690
	8	3	0.0087000	0.00000000	0.00000000	0.0087000	0.0087000	0.00870	0.00870
	9	3	0.0066000	0.00010000	0.00005774	0.0063516	0.0068484	0.00650	0.00670
E (10%)	5	3	0.0022667	0.00005774	0.00003333	0.0021232	0.0024101	0.00220	0.00230
	6	3	0.0014000	0.00000000	0.00000000	0.0014000	0.0014000	0.00140	0.00140
	7	3	0.0059000	0.00017321	0.00010000	0.0054697	0.0063303	0.00580	0.00610
	8	3	0.0045667	0.00015275	0.00008819	0.0041872	0.0049461	0.00440	0.00470
	9	3	0.0032667	0.00011547	0.00006667	0.0029798	0.0035535	0.00320	0.00340

Abbreviations: CI, confidence interval; SD, standard deviation; SE, standard error.

**Table 5:** Raw data for fracture resistance.

Nanosilica	pH					
	Dry	5	6	7	8	9
A (0%)	66.25	14.06	13.86	9.80	15.35	16.08
	65.9	14.28	13.89	9.54	15.49	16.19
	66.6	14.35	13.80	9.76	15.30	16.00
B (2%)	71.1	16.34	16.04	15.20	19.33	21.08
	70.96	16.29	15.97	14.64	19.18	21.13
	70.85	16.12	15.93	14.86	19.24	21.09
C (5%)	101.2	16.62	16.15	16.45	19.49	21.79
	107.4	16.70	16.25	16.20	19.60	22.01
	—	16.98	16.58	16.09	19.65	22.05
D (7%)	82.44	16.72	16.09	16.12	—	21.78
	82.56	16.50	16.29	16.25	19.45	21.66
	81.96	16.43	16.25	16.08	19.23	21.75
E (10%)	55.5	13.24	12.83	12.55	12.02	—
	56.75	13.38	12.99	12.46	12.15	11.81
	56.8	13.28	13.12	12.19	11.92	11.89

**Table 6:** Descriptive statistics and 95% CIs for fracture resistance in different groups.

Variable	Groups	*N*	Mean	SD	SE	95% CI		Minimum	Maximum
Nanosilica	A (0%)	18	22.5833	20.20330	4.76196	12.5365	32.6302	9.54	66.60
	B (2%)	18	26.4083	20.62105	4.86043	16.1537	36.6629	14.64	71.10
	C (5%)	17	28.3065	28.70712	6.96250	13.5466	43.0663	16.09	107.40
	D (7%)	17	29.2682	25.40236	6.16098	16.2075	42.3289	16.08	82.56
	E (10%)	17	20.2871	17.21737	4.17582	11.4347	29.1394	11.81	56.80
pH	Dry	14	74.0193	15.70625	4.19767	64.9508	83.0878	55.50	107.40
	5	15	15.4193	1.44592	0.37333	14.6186	16.2201	13.24	16.98
	6	15	15.0693	1.43593	0.37076	14.2741	15.8645	12.83	16.58
	7	15	13.8793	2.60115	0.67162	12.4389	15.3198	9.54	16.45
	8	14	16.9571	3.13973	0.83913	15.1443	18.7700	11.92	19.65
	9	14	19.0221	3.81705	1.02015	16.8182	21.2260	11.81	22.05

Abbreviations: CI, confidence interval; SD, standard deviation; SE, standard error.

**Table 7:** Descriptive statistics and 95% CIs for fracture resistance in all subgroups.

Nanosilica	pH	*N*	Mean	SD	SE	95% CI		Minimum	Maximum
A (0%)	Dry	3	66.2500	0.35000	0.20207	65.3806	67.1194	65.90	66.60
	5	3	14.2300	0.15133	0.08737	13.8541	14.6059	14.06	14.35
	6	3	13.8500	0.04583	0.02646	13.7362	13.9638	13.80	13.89
	7	3	9.7000	0.14000	0.08083	9.3522	10.0478	9.54	9.80
	8	3	15.3800	0.09849	0.05686	15.1353	15.6247	15.30	15.49
	9	3	16.0900	0.09539	0.05508	15.8530	16.3270	16.00	16.19
B (2%)	Dry	3	70.9700	0.12530	0.07234	70.6587	71.2813	70.85	71.10
	5	3	16.2500	0.11533	0.06658	15.9635	16.5365	16.12	16.34
	6	3	15.9800	0.05568	0.03215	15.8417	16.1183	15.93	16.04
	7	3	14.9000	0.28213	0.16289	14.1991	15.6009	14.64	15.20
	8	3	19.2500	0.07550	0.04359	19.0625	19.4375	19.18	19.33
	9	3	21.1000	0.02646	0.01528	21.0343	21.1657	21.08	21.13
C (5%)	Dry	2	104.3000	4.38406	3.10000	64.9108	143.6892	101.20	107.40
	5	3	16.7667	0.18903	0.10914	16.2971	17.2362	16.62	16.98
	6	3	16.3267	0.22502	0.12991	15.7677	16.8856	16.15	16.58
	7	3	16.2467	0.18448	0.10651	15.7884	16.7049	16.09	16.45
	8	3	19.5800	0.08185	0.04726	19.3767	19.7833	19.49	19.65
	9	3	21.9500	0.14000	0.08083	21.6022	22.2978	21.79	22.05
D (7%)	Dry	3	82.3200	0.31749	0.18330	81.5313	83.1087	81.96	82.56
	5	3	16.5500	0.15133	0.08737	16.1741	16.9259	16.43	16.72
	6	3	16.2100	0.10583	0.06110	15.9471	16.4729	16.09	16.29
	7	3	16.1500	0.08888	0.05132	15.9292	16.3708	16.08	16.25
	8	2	19.3400	0.15556	0.11000	17.9423	20.7377	19.23	19.45
	9	3	21.7300	0.06245	0.03606	21.5749	21.8851	21.66	21.78
E (10%)	Dry	3	56.3500	0.73655	0.42525	54.5203	58.1797	55.50	56.80
	5	3	13.3000	0.07211	0.04163	13.1209	13.4791	13.24	13.38
	6	3	12.9800	0.14526	0.08386	12.6192	13.3408	12.83	13.12
	7	3	12.4000	0.18735	0.10817	11.9346	12.8654	12.19	12.55
	8	3	12.0300	0.11533	0.06658	11.7435	12.3165	11.92	12.15
	9	2	11.8500	0.05657	0.04000	11.3418	12.3582	11.81	11.89

Abbreviations: CI, confidence interval; SD, standard deviation; SE, standard error.

### 3.1 Fracture Resistance

**Figure 2:**
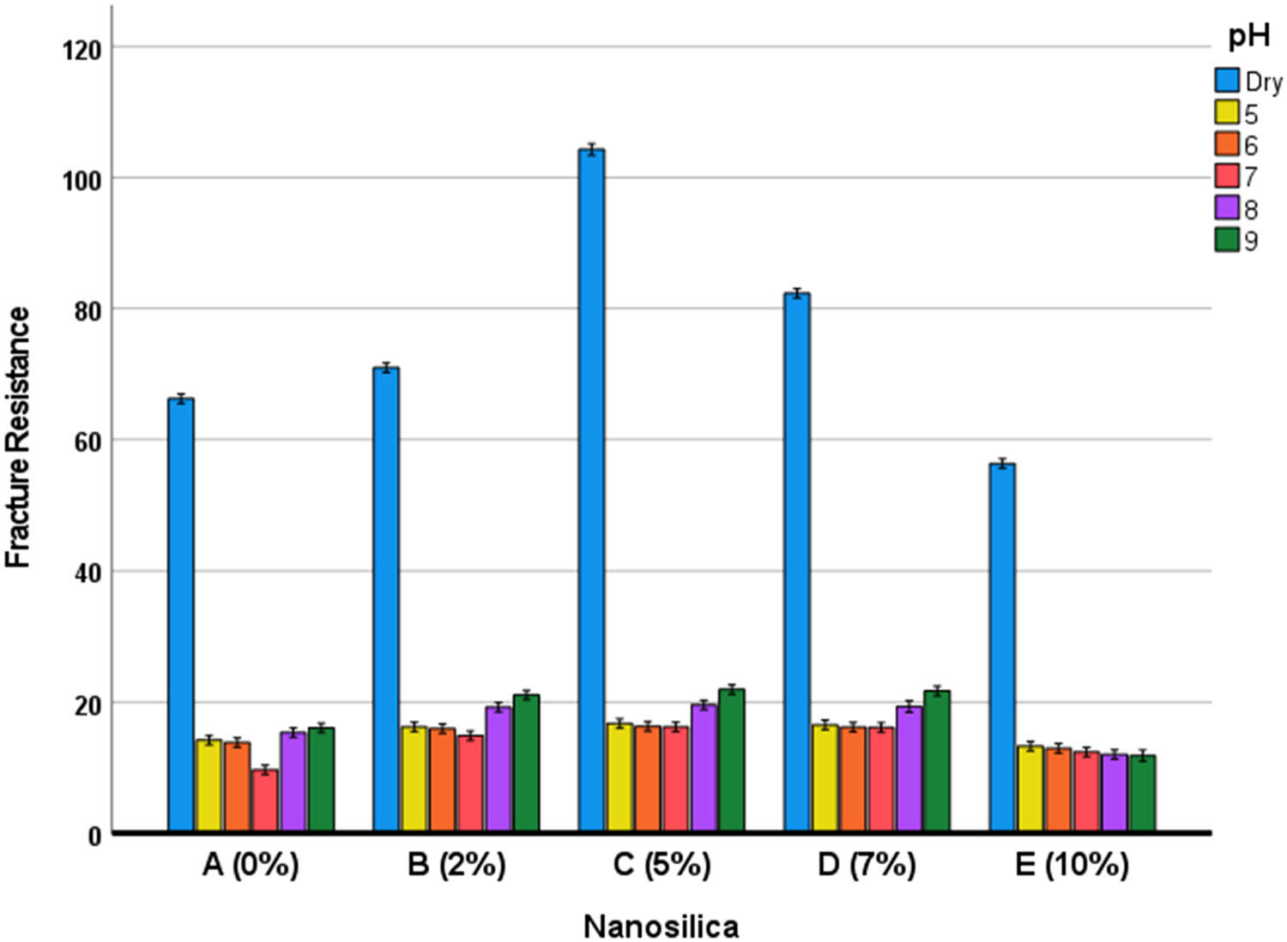
Estimated marginal means and 95% CIs for fracture resistances in different subgroups.

The two‐way ANOVA showed a significant difference between fracture resistances of all nanosilica groups (*p* < 0.00001) and between fracture resistances of all pH groups (*p* < 0.00001). The interaction of nanosilica and pH was ‎significant (*p *< 0.00001). ‎ The Bonferroni test showed significant pairwise comparisons between fracture resistances in different pH groups (including the dry control, Table 8) and different nanosilica groups (Table 9, Figure 2).

**Table 8:** Results of the Bonferroni test, comparing moisture resorption rates and fracture resistances of different pH groups. Note that the dry control group was only relevant to fracture‐resistant testing and not to moisture absorption.

pH groups		Moisture absorption (without dry control, *n* = 75)‎					Fracture resistance (dry control included, *n* = 90)‎				
(I)	(J)	(I−J)	SE	*p*	95% CI		(I−J)	SE	*p*	95% CI	
5	6	−0.004133	0.000102	< 0.000001	−0.004434	−0.003833	0.350	0.225	1.0	−0.340	1.040
	7	−0.015027	0.000102	< 0.000001	−0.015327	−0.014726	1.540	0.225	< 0.000001	0.850	2.230
	8	0.002047	0.000102	< 0.000001	0.001746	0.002347	−1.538	0.229	< 0.000001	−2.240	−0.836
	9	0.003940	0.000102	< 0.000001	0.003639	0.004241	−3.603	0.229	< 0.000001	−4.305	−2.901
6	5	0.004133	0.000102	< 0.000001	0.003833	0.004434	−0.350	0.225	1.0	−1.040	0.340
	7	−0.010893	0.000102	< 0.000001	−0.011194	−0.010593	1.190	0.225	0.000031	0.500	1.880
	8	0.006180	0.000102	< 0.000001	0.005879	0.006481	−1.888	0.229	< 0.000001	−2.590	−1.186
	9	0.008073	0.000102	< 0.000001	0.007773	0.008374	−3.953	0.229	< 0.000001	−4.655	−3.251
7	5	0.015027	0.000102	< 0.000001	0.014726	0.015327	−1.540	0.225	< 0.000001	−2.230	−0.850
	6	0.010893	0.000102	< 0.000001	0.010593	0.011194	−1.190	0.225	0.000031	−1.880	−0.500
	8	0.017073	0.000102	< 0.000001	0.016773	0.017374	−3.078	0.229	< 0.000001	−3.780	−2.376
	9	0.018967	0.000102	< 0.000001	0.018666	0.019267	−5.143	0.229	< 0.000001	−5.845	−4.441
8	5	−0.002047	0.000102	< 0.000001	−0.002347	−0.001746	1.538	0.229	< 0.000001	0.836	2.240
	6	−0.006180	0.000102	< 0.000001	−0.006481	−0.005879	1.888	0.229	< 0.000001	1.186	2.590
	7	−0.017073	0.000102	< 0.000001	−0.017374	−0.016773	3.078	0.229	< 0.000001	2.376	3.780
	9	0.001893	0.000102	< 0.000001	0.001593	0.002194	−2.065	0.233	< 0.000001	−2.779	−1.351
9	5	−0.003940	0.000102	< 0.000001	−0.004241	−0.003639	3.603	0.229	< 0.000001	2.901	4.305
	6	−0.008073	0.000102	< 0.000001	−0.008374	−0.007773	3.953	0.229	< 0.000001	3.251	4.655
	7	−0.018967	0.000102	< 0.000001	−0.019267	−0.018666	5.143	0.229	< 0.000001	4.441	5.845
	8	−0.001893	0.000102	< 0.000001	−0.002194	−0.001593	2.065	0.233	< 0.000001	1.351	2.779
Dry	5						58.600	0.229	< 0.000001	57.898	59.302
	6						58.950	0.229	< 0.000001	58.248	59.652
	7						60.140	0.229	< 0.000001	59.438	60.842
	8						57.062	0.233	< 0.000001	56.348	57.776
	9						54.997	0.233	< 0.000001	54.283	55.711

Abbreviations: CI, confidence interval; SE, standard error.

**Table 9:** Results of the Bonferroni test, comparing moisture absorptions and fracture resistances of different nanosilica groups.

Nanosilica groups		Moisture absorption (without dry control, *n* = 75)					Fracture resistance (dry control included, *n* = 90)				
(I)	(J)	(I−J)	SE	*p*	95% CI		(I−J)	SE	*p*	95% CI	
A (0%)	B (2%)	0.012047	0.000102	< 0.000001	0.011746	0.012347	−3.825	0.206	< 0.000001	−4.425	−3.225
	C (5%)	0.014860	0.000102	< 0.000001	0.014559	0.015161	−5.723	0.209	< 0.000001	−6.332	−5.114
	D (7%)	0.015980	0.000102	< 0.000001	0.015679	0.016281	−6.685	0.209	< 0.000001	−7.294	−6.076
	E (10%)	0.025107	0.000102	< 0.000001	0.024806	0.025407	2.296	0.209	< 0.000001	1.687	2.905
B (2%)	A (0%)	−0.012047	0.000102	< 0.000001	−0.012347	−0.011746	3.825	0.206	< 0.000001	3.225	4.425
	C (5%)	0.002813	0.000102	< 0.000001	0.002513	0.003114	−1.898	0.209	< 0.000001	−2.507	−1.289
	D (7%)	0.003933	0.000102	< 0.000001	0.003633	0.004234	−2.860	0.209	< 0.000001	−3.469	−2.251
	E (10%)	0.013060	0.000102	< 0.000001	0.012759	0.013361	6.121	0.209	< 0.000001	5.512	6.730
C (5%)	A (0%)	−0.014860	0.000102	< 0.000001	−0.015161	−0.014559	5.723	0.209	< 0.000001	5.114	6.332
	B (2%)	−0.002813	0.000102	< 0.000001	−0.003114	−0.002513	1.898	0.209	< 0.000001	1.289	2.507
	D (7%)	0.001120	0.000102	< 0.000001	0.000819	0.001421	−0.962	0.212	0.000289	−1.579	−0.344
	E (10%)	0.010247	0.000102	< 0.000001	0.009946	0.010547	8.019	0.212	< 0.000001	7.402	8.637
D (7%)	A (0%)	−0.015980	0.000102	< 0.000001	−0.016281	−0.015679	6.685	0.209	< 0.000001	6.076	7.294
	B (2%)	−0.003933	0.000102	< 0.000001	−0.004234	−0.003633	2.860	0.209	< 0.000001	2.251	3.469
	C (5%)	−0.001120	0.000102	< 0.000001	−0.001421	−0.000819	0.962	0.212	0.000289	0.344	1.579
	E (10%)	0.009127	0.000102	< 0.000001	0.008826	0.009427	8.981	0.212	< 0.000001	8.363	9.599
E (10%)	A (0%)	−0.025107	0.000102	< 0.000001	−0.025407	−0.024806	−2.296	0.209	< 0.000001	−2.905	−1.687
	B (2%)	−0.013060	0.000102	< 0.000001	−0.013361	−0.012759	−6.121	0.209	< 0.000001	−6.730	−5.512
	C (5%)	−0.010247	0.000102	< 0.000001	−0.010547	−0.009946	−8.019	0.212	< 0.000001	−8.637	−7.402
	D (7%)	−0.009127	0.000102	< 0.000001	−0.009427	−0.008826	−8.9812	0.21152	< 0.000001	−9.599	−8.363

### 3.2 Moisture Absorption

**Figure 3:**
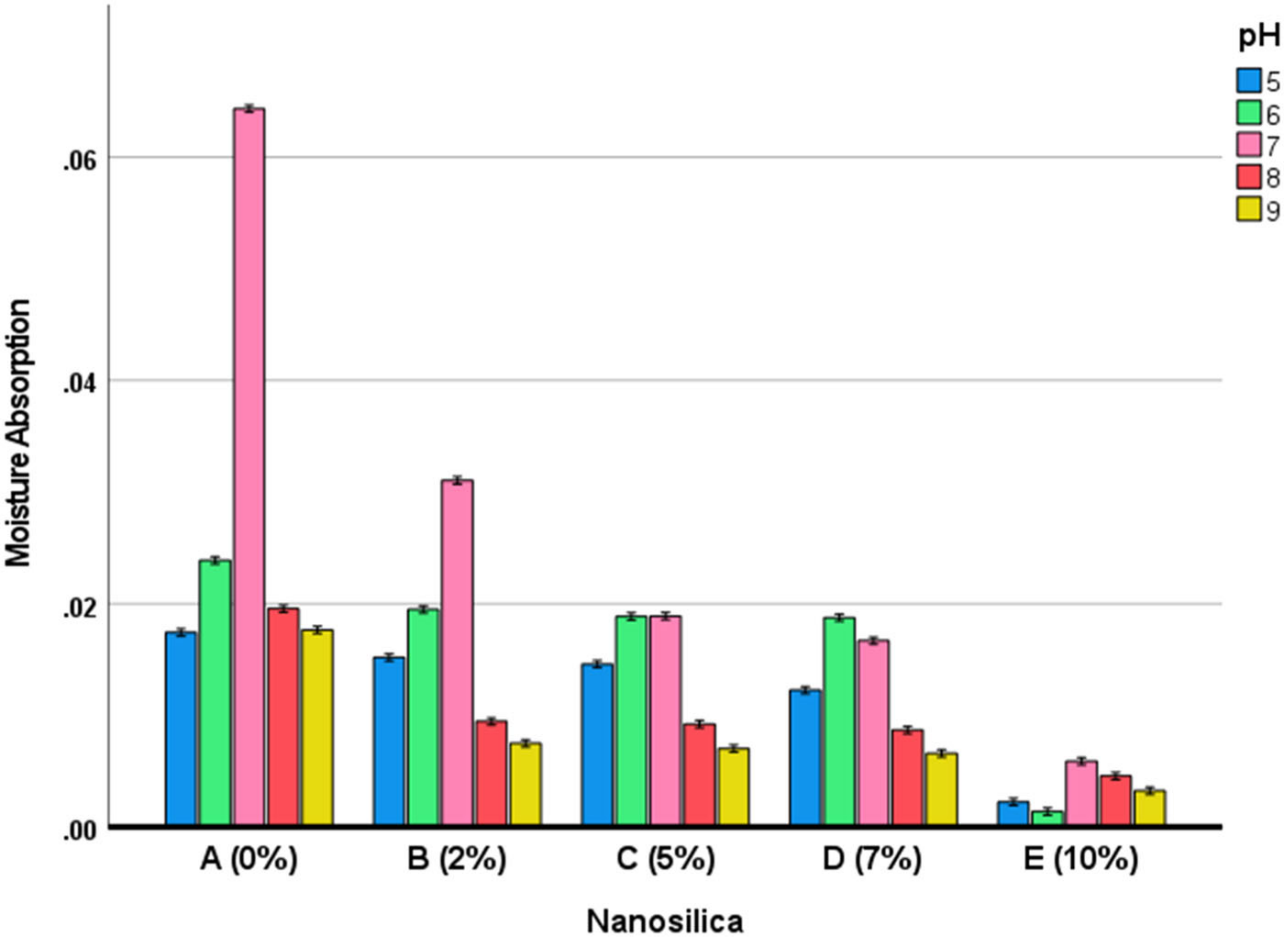
Estimated marginal means and 95% CIs for moisture absorption in different subgroups.

Two‐way ANOVA identified both the variables nanosilica (*p* < 0.00001) and pH (*p* < 0.00001). The interaction of nanosilica and pH was significant as well (*p *< 0.00001). The Bonferroni test showed significant pairwise comparisons between moisture absorptions in different pH groups (Table 8) and different nanosilica groups (Table 9, Figure 3).

### 3.3 Correlation

The Pearson's coefficient showed a significant negative correlation between fracture toughness and moisture absorption (*R* = −0.382, *p* = 0.0009).

### 3.4 SEM

**Figure 4:**
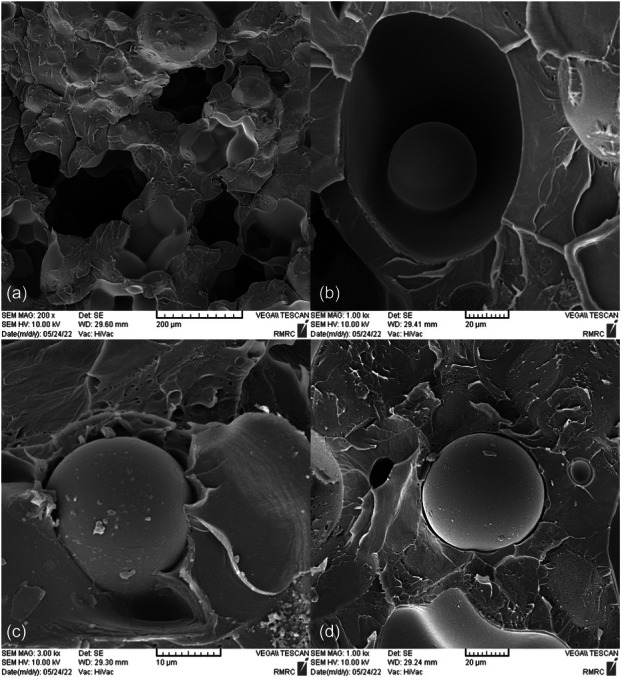
An SEM example of a specimen; (a and b) cavities created as a result of moisture absorption; and (c and d) swelling of the base polymer as a result of moisture absorption.

**Figure 5:**
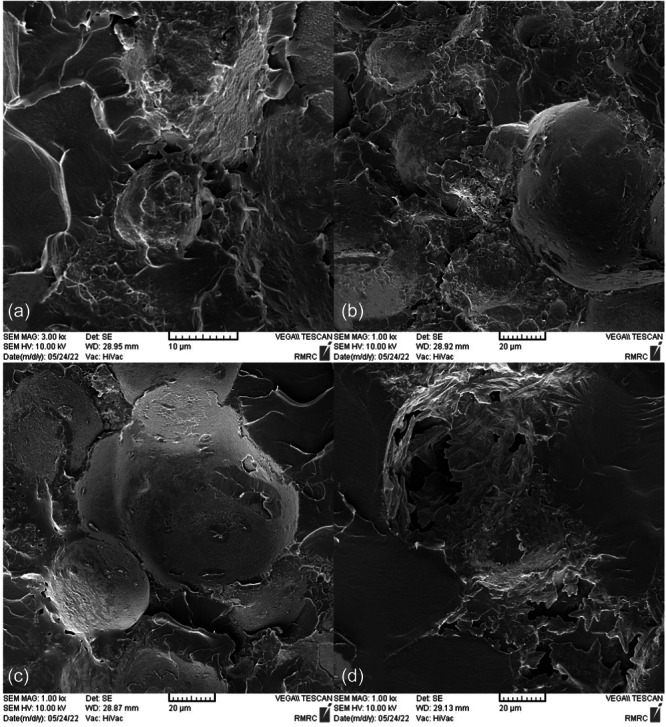
An SEM example of a specimen; (a) continuity of base polymer with the silica filler phase; (b and c) swelling of the base polymer as a result of moisture absorption; and (d) discontinuity of silica nanoparticles at the place of formation of agglomerates.

**Figure 6:**
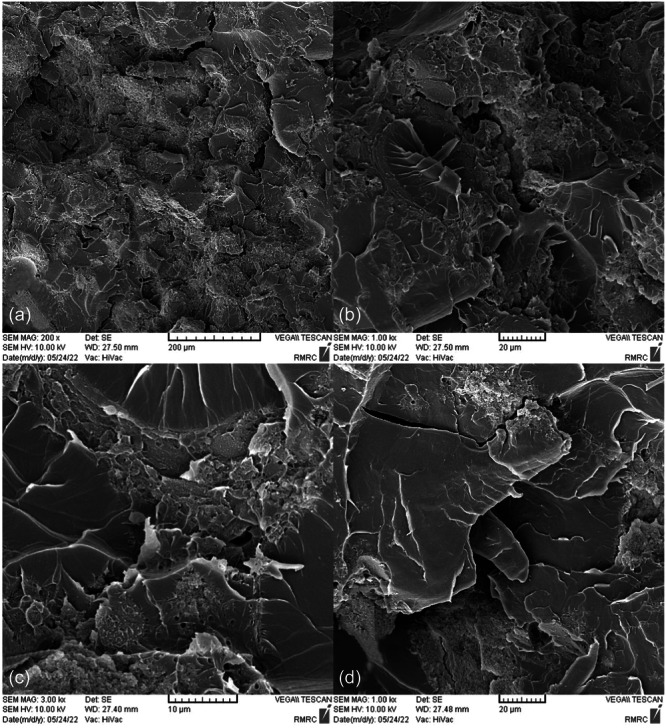
An SEM example of a specimen. (a) Uniform dispersion of silica nanoparticles on the base polymer surface; (b) nonuniform dispersion of silica nanoparticles on the base polymer surface; (c) presence of voids due to air entrapment during mixing and mold filling; and (d) crack formation around holes caused by moisture absorption.

**Figure 7:**
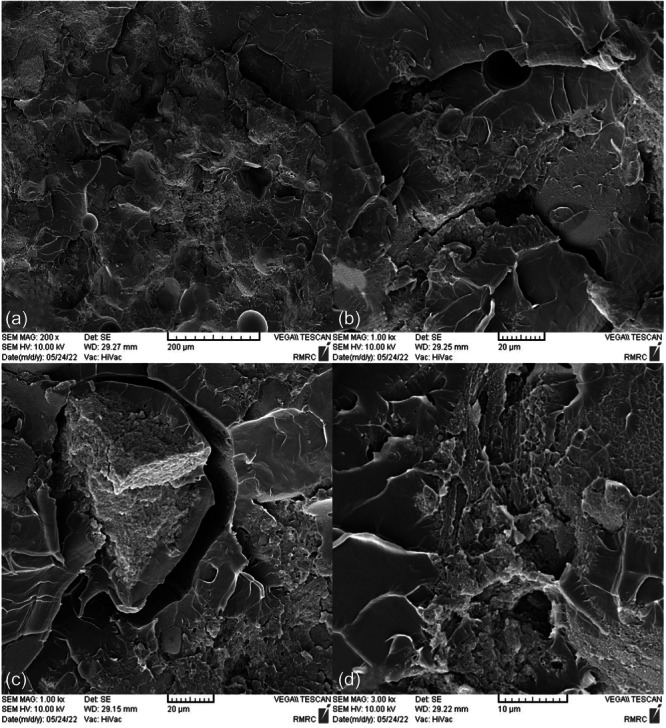
An SEM example of a specimen. (a) Uniform dispersion of silica nanoparticles on the base polymer surface; (b) cavities and cracks created as a result of moisture absorption; (c) creation of cracks around the place of formation of agglomerates; and (d) nonuniform dispersion of silica nanoparticles on the base polymer surface.

**Figure 8:**
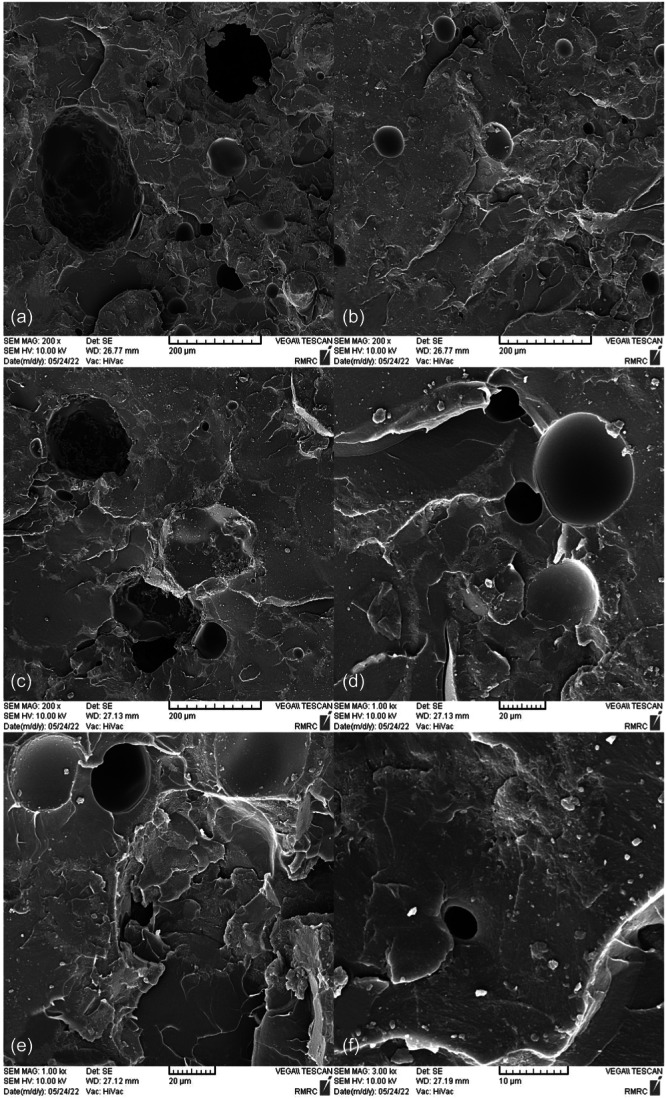
An SEM example of a specimen; (a, c, and d) cavities and cracks created as a result of moisture absorption; (b) presence of voids due to air entrapment during mixing and mold filling; and (e and f) nonuniform dispersion of silica nanoparticles on the base polymer.

**Figure 9:**
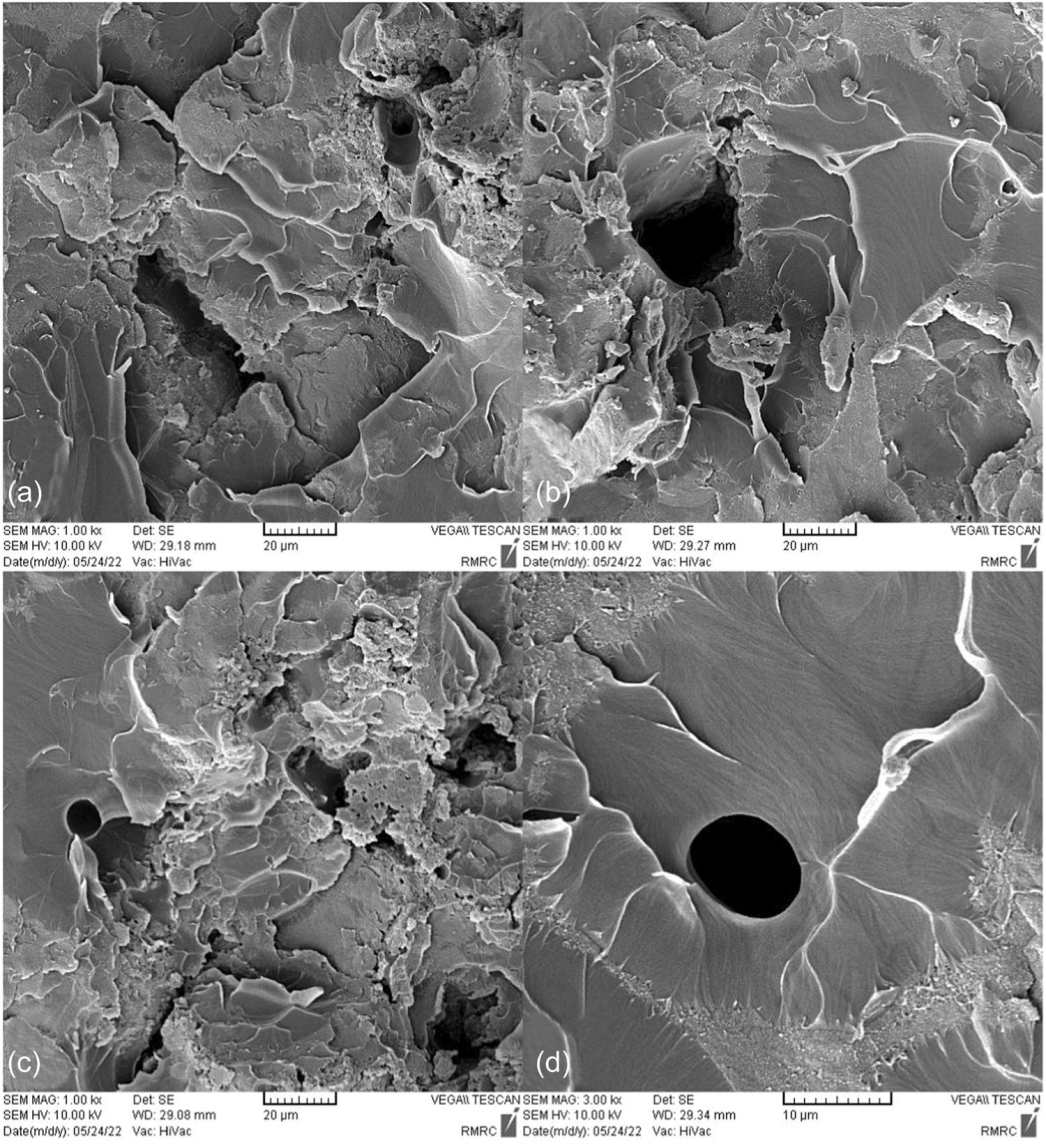
An SEM example of a specimen; (a−c) nonuniform dispersion of silica nanoparticles on the base polymer surface and creation of cracks around the place of formation of agglomerates; and (d) cavities created as a result of moisture absorption.

SEM images taken from the surface of samples show that moisture absorption causes cavities and swelling in the base polymer, and the ‎samples break as a result of the creation and expansion of cracks that are created around these ‎cavities (Figures 4, 5, 6, 7, 8, 9). SEM images showed that up to 7 wt% of the filler, the dispersion of silicon particles was good, but for the case where 10 wt% of the filler was used, the dispersion of the particles was not uniform.

## 4 Discussion

As there was no study similar to our explorative research, we are limited to discussion of more general aspects of the study. The literature does not contain adequate studies in this regard, and the existing ones are about scattered and different aspects and properties of this field, not to mention that they are controversial at points. Zhen et al. ‎ (2020) and Balos et al. ‎ (2020) found that by increasing silica NPs from 0% to 5%, the tensile strength and elongation at the breaking point of the resins improved. Also, the bending strength and elastic modulus increased significantly. Fatalla, Tukmachi, and Jani (2020) observed a slight increase in impact strength, transverse strength, and hardness with a negligible amount of 5% wt of nanosilica. Siot et al. (2019) asserted that although silica NPs increase the static modulus and yield strength of resins, the final properties of the resin are strongly affected by the dispersion properties of the NPs, such that a better performance of the silica filler is related to its regular dispersion. Evaluating the effect of mixing modified and unmodified silica NPs, Mussatto et al. ‎ (2020) concluded that by adding silica NPs, the bending strength of the resin in both modified and unmodified states decreased by 20%−27%; this reduction was not dependent on the amount of filler and the method of combining NPs. Moreover, they found that the surface modification of nanosilica did not cause any major improvement in the mechanical behavior of nano‐resins, but it seemed to improve the dispersion because the surface roughness was smaller. Salman, Jani, and Fatalla (2017) observed very significant improvements in impact strength, transverse strength, and stiffness by adding nanosilica to PMMA at 3%, 5%, and 7% by weight. They concluded that the addition of NPs in the optimal state could yield better physical and mechanical properties. Topouzi et al. (2017) observed a significant increase in fracture toughness by adding modified and unmodified silica NPs to PMMA; nevertheless, they concluded that increasing the concentration of the filler decreased the fracture toughness. Also, they did not report any significant difference between modified and unmodified fillers. Kundie, Azhari, Muchtar, et al. ‎ (2018) concluded that the effect of NPs on the mechanical properties of dental resins strongly relied on factors such as the type and mechanical properties of mineral nanofillers, the uniform dispersion of nanofillers in the polymer matrix, the volume of the filler particles, and the type of silane used.

In the present study, polymeric resins filled with different percentages of hard mineral particles in nano dimensions showed different behaviors with increasing percentage of particles. Polymer water absorption is the result of molecular polarity, unsaturated bonds of molecules, and unbalanced intermolecular forces in polymers. According to the ISO 1567 standard, water absorption for thermal and self‐baking materials should not exceed 32 mg/mm^3^ (Karabela and Sideridou 2011). According to our results, with increasing percentage of nanosilica, the amount of moisture absorption decreases. The reason for this could be related to the amorphous structure of the background polymer. The distance between PMMA molecules in the absence of silica filler particles was greater than the matrix to which NPs were added. Silica NPs fill the space between molecules, so the more the amount of nanosilica in the base polymer, the smaller the distance between molecules, and more regular the structure of the polymer, and hence a decrease in the amount of moisture absorption (Zirak et al. 2019). The results of the experiments also showed the lowest amount of moisture absorption at 10 wt% of nanosilica (Zirak et al. 2019).

Furthermore, by comparing the findings obtained at different pH levels, it was observed that the more neutral the pH of the liquid bath, the higher the absorption rate. The highest amount of moisture absorption was observed at pH = 7. The chemical structure of PMMA shows that the background polymer atoms do not tend to react with acidic and basic solvents, and as a result, the amount of absorption in these environments decreases. Also, silica NPs that were in the base polymer did not tend to react with acids and bases (Ilie et al. 2017). Therefore, it was expected that as the liquid became acidic or basic, the amount of absorption decreased.

Studies show that the main problem with using silica NPs in dental resins is the accumulation of silica NPs next to each other when combined with PMMA, which leads to the formation of micrometer particles, and this reduces the mechanical properties of resins enriched with NPs (Siot et al. 2019; Kundie, Azhari, Muchtar, et al. 2018). Therefore, surface modification of the filler particles is needed to reduce the formation of agglomerates and achieve a better distribution of the filler particles inside the matrix (Mussatto et al. 2020).

Our findings indicated that increasing the amount of silica filler increased the fracture toughness of the samples. However, when the amount of filler was more than 5 wt%, not only did the fracture toughness not improve but also it began to decrease. Thus, in the case of 10 wt% nanosilica, the fracture resistance of these samples was lower than that of samples without silica filler (Balos et al. 2020). As polymers have an amorphous and irregular structure, increasing the filler particles reduces the distance between the particles, which reduces the crack propagation rate. Increasing the length of the crack propagation path increases the fracture toughness of dental resin. Smaller sizes of the filler particles increase the surface energy created at the junction of the resin and the filler, and this improves flexural strength. The addition of NPs also improved the hardness because solid mineral particles usually have higher hardness compared to polymeric matrices. Researchers believe that the reason for the increase in the fracture toughness of resins by addition of filler is due to the interaction of the crack tip with the filler phase. Moreover, increasing the amount of filler increases the accumulation of particles and causes the formation of agglomerates. The formation of these points accelerates crack growth and decreases fracture toughness (Kundie, Azhari, Muchtar, et al. 2018). Although in this research, an attempt was made to create a more uniform distribution of silica NPs on the surface of the base polymer by modifying the filler particles, in high weight percentages, the accumulation of NPs created agglomerates. Therefore, the fracture toughness of resins is a complex process, and its increase or decrease cannot be attributed only ‎to the concentration of fillers. The study on the mechanism of failure in resins shows that the type of filler, the process of failure, the protrusion and thickness of the crack tip, the plastic yield limit of the matrix, the structure of the resin, the separation of the interface between the particles and the matrix, and especially the accumulation of filler particles are among other factors that can affect the test results of the fracture toughness of resins. Also, by examining the fracture toughness of samples that were in a humid environment, it was found that moisture absorption caused a significant decrease in the fracture toughness of dental samples. As the amount of moisture absorption increased, the fracture toughness decreased. Hence, at pH = 7, which had the highest moisture absorption, the greatest decrease in failure toughness was observed. SEM images showed that moisture absorption caused the formation of microcracks in the base polymer, and this led to crack growth and, as a result, reduced fracture toughness of the dental samples. Furthermore, PMMA swelled slightly by absorbing water, which is different from the polymer composition of the particle components, and this weakened the mechanical properties, including the fracture toughness of the dental samples (Karabela and Sideridou 2011; Zirak et al. 2019).

## 5 Conclusion

It can be concluded that

1. Increasing the amount of silica filler improves the fracture toughness, but there is an optimal limit (5 wt% nanosilica filler).
2. Increasing the percentage of nanosilica filler leads to the accumulation of nanosilica particles and the formation of agglomerates.
3. Increasing the nanosilica filler concentration reduces moisture absorption.
4. The tested samples react to acidic and alkaline environments. Accordingly, the more the acidity or alkalinity of the liquid becomes neutral, the more the absorption of moisture in the dental samples increases.
5. Also, in samples with less than 10% filler weight, the amount of moisture absorption in acidic environments is higher than that in alkaline environments, but at 10 wt% of the filler, the amount of moisture absorption in an alkaline environment is higher compared to an acidic environment.
6. With an increase of moisture absorption, the fracture toughness of dental samples decreases significantly.

## Author Contributions

All the authors meet the ICMJE criteria for authorship. Nima Refahati and Mohammad Ali Golshokouh made substantial contributions to the conception or design, acquisition, analysis, and interpretation of data. Mohammad Ali Golshokouh, Nima Refahati, and Pouyan Roodgar Saffari drafted and critically reviewed the work for important intellectual content, and approved the final version. All authors agreed to be accountable for all aspects of the work, ensuring that questions related to the accuracy or integrity of any part of the work are appropriately investigated and resolved.

## Conflicts of Interest

The authors declare no conflicts of interest.

## Data Availability

The data are available from the corresponding author.
